# Sheep Genetic Resistance to Gastrointestinal Nematode Infections: Current Insights from Transcriptomics and Other OMICs Technologies—A Review

**DOI:** 10.3390/pathogens15010106

**Published:** 2026-01-19

**Authors:** Krishani Sinhalage, Guilherme Henrique Gebim Polizel, Niel A. Karrow, Flavio S. Schenkel, Ángela Cánovas

**Affiliations:** 1 Centre for Genetic Improvement of Livestock, Department of Animal Biosciences, University of Guelph, 50 Stone Road East, Guelph, ON N1G 2W1, Canada; ksinhala@uoguelph.ca (K.S.); or guilherme.polizel@usp.br (G.H.G.P.); nkarrow@uoguelph.ca (N.A.K.); schenkel@uoguelph.ca (F.S.S.); 2 Department of Animal Science, Faculty of Animal Science and Food Engineering, University of São Paulo, Av. Duque de Caxias Norte, 225, Pirassununga 13635-900, Brazil

**Keywords:** GIN resistance, multi-omics, genomics, metabolomics, proteomics, microbiome

## Abstract

Gastrointestinal nematode (GIN) infections are the most prevalent parasitic diseases in grazing sheep worldwide, causing significant productivity losses, high mortality and, as a result, economic losses and emerging animal welfare concerns. Conventional control strategies, primarily relying on anthelmintic treatments, face limitations due to rising drug resistance and environmental concerns, underscoring the need for sustainable alternatives. Selective breeding for host genetic resistance has emerged as a promising strategy, while recent advances in transcriptomics and integrative omics research are providing deeper insights into the immune pathways and molecular and genetic mechanisms that underpin host–parasite interactions. This review summarizes current evidence on transcriptomic signatures associated with resistance and susceptibility to *H. contortus* and *T. circumcincta* GIN infections, highlighting candidate genes, functional genetic markers, key immune pathways, and regulatory networks. Furthermore, we discuss how other omics approaches, including genomics, proteomics, metabolomics, microbiome, and multi-omics integrations, provide perspectives that enhance the understanding of the complexity of the GIN resistance trait. Transcriptomic studies, particularly using RNA-Sequencing technology, have revealed differential gene expression, functional genetic variants, such as SNPs and INDELs, in expressed regions and splice junctions, and regulatory long non-coding RNAs that distinguish resistance from susceptible sheep, highlighting pathways related to Th2 immunity, antigen presentation, tissue repair, and stress signaling. Genomic analyses have identified SNPs, QTL, and candidate genes linked to immune regulation and parasite resistance. Proteomic and metabolomic profiling further elucidates breed- and tissue-specific alterations in protein abundance and metabolic pathways, while microbiome studies demonstrate distinct microbial signatures in resistant sheep, suggesting a role in modulating host immunity. In conclusion, emerging multi-omics approaches and their integration strategies provide a comprehensive framework for understanding the complex host–parasite interactions that govern GIN resistance, offering potential candidate biomarkers for genomic selection and breeding programs aimed at developing sustainable, parasite-resistant sheep populations.

## 1. Introduction

Gastrointestinal nematode (GIN) infections are one of the most prevailing parasitic diseases in grazing sheep worldwide [1], resulting in loss of sheep productivity, high mortality rate, subsequent economic losses [2,3] and emerging animal welfare issues [4]. Common GIN species include *Haemonchus contortus* (*H. contortus*), *Teladorsagia circumcincta* (*T. circumcincta*), *Trichostrongylus* spp., and *Oesophagostomum* spp. [5,6] with mixed infections frequently occurring in grazing systems across temperate and tropical regions [4,7]. However, the most prevalent species may vary depending on the geographical location and climate [8,9,10]. Among these, *H. contortus* is considered the most pathogenic endoparasite that causes haemonchosis, a leading parasitic disease in small ruminants worldwide [7,10,11].

Infection by GINs in the abomasum and small intestine of both young and adult sheep leads to significant health and welfare issues [11]. Clinical signs include digestive tract inflammation, disruption of gastrointestinal integrity, and nutrient malabsorption [12]. These pathological effects contribute to diarrhea, hypoproteinemia, reduced feed efficiency, progressive weight loss, poor growth, loss of body condition, decreased reproductive performance, and, in severe cases, death [6,7,13,14,15]. Moreover, GIN infections often cause varying degrees of anemia due to the blood-feeding nature of parasites. Even subclinical infections can have detrimental effects on production traits such as wool, milk and meat quality, while simultaneously increasing management and veterinary costs [7].

Conventional GIN control strategies primarily rely on the use of anthelmintic treatments in combination with grazing management practices, both of which incur substantial costs [16,17,18]. However, improper and frequent use of anthelmintics has resulted in widespread anthelmintic resistance within parasite populations [3,19,20,21]. Additionally, excessive use of anthelmintics contributes to the accumulation of drug residues in food and the environment [16,22]. Hence, the development of sustainable and alternative strategies for controlling GIN infections in sheep has become a pressing need worldwide.

Genetic resistance, the innate ability of certain sheep to limit parasite establishment, fecundity, or pathogenesis, has emerged as a promising solution, reducing reliance on veterinary drugs [21,23]. Selection for natural resistance, particularly through reduced fecal egg counts (FEC) as an indirect measure, lowers the number of eggs shed into the environment, thereby decreasing pasture contamination and exposure of susceptible animals [19,21,24]. Heritability estimates for FEC typically range from 0 to 0.4 [6], indicating moderate genetic control of this trait across breeds and production systems [25]. However, genetic resistance to GIN infections is a complex, polygenetic trait [3], influenced by multiple biological pathways and immune mechanisms [25].

With the advent of high-throughput sequencing and other omics platforms, our understanding of complex traits shaped by the aggregate effects of many genes or loci and modulated by varied environmental conditions has been transformed. Integration of different types of omics data, including transcriptomics, genomics, proteomics, metabolomics, and microbiomics, enables the direct and systematic characterization of the molecular basis of host–parasite interactions and immune responses to parasitic infections [26,27], while also enabling the identification of potential biomarkers associated with complex traits [28]. Because genetic variation, gene expression, protein function, metabolic state, and host-associated microbial communities jointly shape host responses to infection, no single omics approach fully captures the multifactorial and context-dependent nature of GIN resistance. Consequently, integrative multi-omics strategies are increasingly recognized as essential for linking molecular mechanisms to resistance phenotypes [29] and for informing genomic selection and precision breeding programs aimed at improving GIN resistance in sheep.

Transcriptomics allows for the systematic profiling of the complete set of RNA expressions within specific cells, tissues, or organs at a given time point, capturing dynamic host responses following GIN challenge [27,30]. Studies of the abomasal mucosa and lymph nodes of infected sheep using RNA-Sequencing technology (RNA-Seq) have consistently shown that GIN infection elicits a canonical T helper 2 (Th2) driven immune response that distinguishes resistant from susceptible sheep [31,32]. Genomics enables the analysis of DNA variation underlying susceptibility or resistance, providing insights into the genetic architecture and functional variants involved in immune defense [27,30]. Proteomics extends this understanding by quantifying protein abundance and associated signaling pathways, providing a comprehensive profile of the protein complement within a cell, fluid, tissue, or organism at a specific time point [30,33]. Conversely, metabolomics identifies and quantifies metabolites, thereby revealing changes in metabolic processes linked to the host’s physiological and immunological adaptation to infection [30].

Moreover, the gastrointestinal microbiome plays a crucial role in modulating host responses to GIN infections. Resistant sheep exhibit distinct, more diverse microbial communities enriched with carbohydrate-fermenting taxa, which may contribute to parasite inhibition and improved gut health [34]. Infection-induced shifts in microbial composition have also been linked to pro-inflammatory responses and altered metabolism, further highlighting the interplay between the microbiome, host immunity, and parasite dynamics [35,36].

Given the complexity of host–parasite interactions and the multifaceted biological functions that influence resistance, an integrated understanding across transcriptomics, genomics, metabolomics, proteomics, and microbiomics is essential. Therefore, this review aims to synthesize current omics findings and emerging multi-omics integration efforts to elucidate the molecular and genetic mechanisms underlying resistance to GINs, specifically *H. contortus* and *T. circumcincta* in sheep, highlighting key pathways, candidate genes, proteomic, metabolomic, and microbial signatures associated with resistant phenotypes. By evaluating insights from individual omics platforms as well as emerging integrative approaches, this review provides a comprehensive framework to inform future research and accelerate the development of effective genomic selection and sustainable breeding strategies for parasite-resistant sheep.

## 2. Literature Search Strategy and Information Sources

This review is based on a structured survey of peer-reviewed literature identified through targeted searches of electronic databases, including Web of Science, PubMed, Science Direct, and Google Scholar, focusing on genetic resistance of sheep to GIN infections. Searches, last conducted in November 2025, emphasized host genetic resistance, immune responses, and molecular mechanisms underlying GIN infections, with a particular focus on omics-based approaches, including transcriptomics, genomics, proteomics, metabolomics, microbiome studies and integration of multi-omics technologies. Priority was given to peer-reviewed original research articles published primarily from 2015 onward to capture recent advances in RNA sequencing and multi-omics approaches during this period. Reference lists of key articles were manually screened to identify additional relevant studies. Evidence from non-ovine species was included only where it provided a supportive biological context.

### 2.1. Search Scope and Keywords

To address the multidisciplinary scope of genetic resistance to GIN infections, database searches combined the core terms “sheep” OR “ovine” with “gastrointestinal nematodes” and “genetic resistance”, alongside topic-specific keywords corresponding to the main themes of the review. These included transcriptomics, RNA-Seq, differential expression of genes, messenger RNA (mRNA) isoforms, long non-coding RNAs (lncRNAs), functional single nucleotide polymorphisms (SNPs), insertions and deletions (INDELs), genomics, genome-wide association studies (GWAS), quantitative trait loci (QTL), proteomics, metabolomics, microbiome, immune response, and multi-omics integration. Searches also included parasite-specific terms such as *H. contortus* and *T. circumcincta*, reflecting their prominence in resistance studies.

### 2.2. Study Selection Rationale and Limitations

Studies were selected based on their relevance to host genetic, transcriptomic, proteomic, metabolomic, or microbiome-associated mechanisms underlying resistance or susceptibility to GIN infections in sheep. As a thematic review, study selection was guided by relevance rather than a formal systematic review protocol, and potential selection bias is acknowledged. Limitations include restrictions to English-language publications, heterogeneity in experimental designs, infection models, resistance phenotypes (e.g., fecal egg count, immune markers), geographic and production systems, and uneven breed representation rather than targeted prioritization.

## 3. Transcriptional Dynamics in Host–Parasite Interactions

### 3.1. Overview of Transcriptomics

Transcriptomics provides a comprehensive analysis of the transcriptome, investigating transcription and regulation of both mRNAs and non-coding RNAs (ncRNA) expressed within a cell, tissue, or organ at a given time point. ncRNAs, which include microRNAs (miRNAs), lncRNAs, and circular RNAs (circRNAs), are particularly important as key regulators of mRNA transcription initiation and post-transcriptional modification [30].

In the context of GIN infections, transcriptomic analyses enable the discovery of candidate genes, metabolic pathways, and functional and structural genetic variants that underlie host immune responses [23]. By examining the spatiotemporal expression of genes in key tissues, such as the abomasal mucosa and lymph nodes, transcriptomics provides insights into the molecular mechanisms driving resistance and susceptibility in sheep [32,37,38,39,40].

Transcriptome analysis has evolved from low-throughput, candidate gene-based methods to high-throughput global approaches. Early studies employed Northern blotting, which required large amounts of RNA and radioactive probes, which was later replaced by reverse transcription–polymerase chain reaction (RT-PCR), allowing more sensitive and efficient quantification of gene expression [41]. The development of microarray technology enabled simultaneous measurement of thousands of transcripts in a cost-effective manner, though it is limited by cross-hybridization, inability to detect novel transcripts, and relative quantification [30,41]. More recently, RNA-Seq has revolutionized transcriptomics by providing a high-throughput, quantitative, and genome-wide assessment of gene expression [42,43,44], with the added ability to detect novel transcripts, alternative splicing isoforms [30,41], functional genetic variants, such as SNPs and INDELs within expressed regions [45,46,47,48,49,50], and ncRNAs, including lncRNAs [51], overcoming the main limitations of microarrays and PCR-based methods [30,41].

### 3.2. RNA-Seq Approaches to Host Immune Responses Against GIN Infections

In the context of ovine GIN infections, RNA-Seq is instrumental for comparing the tissue-specific immune responses of resistant and susceptible sheep. By analyzing transcriptomic profiles from key sites of infection, such as the abomasal mucosa, lymph nodes, and small intestine, researchers can identify the differentially expressed genes, novel transcripts, functional and structural variants [3,4,23,25,32,37,38,39,50], and regulatory ncRNAs that modulate the host defense mechanisms against GIN infections [51].

This section of the review focuses on RNA-Seq-based transcriptomic studies that have investigated differential gene expression, mRNA isoforms, lncRNAs, and functional and structural genetic variants, including SNPs and INDELs, in resistant and susceptible sheep in response to GIN infections. As many previous studies have examined *H. contortus* and *T. circumcincta* infections in relation to ovine genetic resistance, we primarily focus on these two nematode species.

#### 3.2.1. Differential Gene Expression

RNA-Seq has been widely used to identify differentially expressed genes (DEGs) in sheep with varying immune responses to GIN infections. Experimental design is a crucial component in RNA-Seq research, as it strongly influences how transcriptomic data are interpreted [38,52]. Most studies included in this review compare resistant and susceptible sheep, and such comparative approaches are essential for identifying DEGs and immune-related pathways underlying variation in GIN resistance.

This review synthesizes RNA-Seq studies published between 2015 and 2025 to provide an updated overview of transcriptomic insights into the host responses to *H. contortus* and *T. circumcincta* GIN infections in sheep. These studies encompass a range of sheep breeds representing diverse production systems and geographical regions, including Canaria Hair [53,54], Canaria [53,54], Scottish Blackface [38], Churra [32], Merino [39,55,56], St. Croix [57], Suffolk [57], Santa Ines [58], Ile de France [58], and Rideau x Dorset crosses [4,23]. Conducted across Europe, Australia, China, the USA, Canada, and Brazil, these investigations collectively demonstrate that host genetic background strongly influences transcriptional responses to GIN infections. Comparisons between resistant and susceptible animals within and across breeds reveal both shared immune pathways and breed-specific transcriptomic signatures, highlighting the importance of breed context when interpreting RNA-Seq findings related to parasite resistance.

Sampling time points are critical in transcriptomic studies, as they determine the ability to detect key immune responses, with early post-infection sampling often capturing innate immune activation, whereas later stages reflect adaptive and regulatory responses [52,59]. The type of tissue sampled also influences the findings. The studies summarized in this review have analyzed a range of tissues, including abomasal mucosa, abomasal lymph nodes, peripheral blood, and liver, to capture both local and systemic host immune responses. Abomasal mucosa provides direct insights into host–parasite interactions at the infection site, while abomasal lymph nodes offer valuable information on adaptive immune responses [52,60]. In contrast, liver tissue used in some studies can reflect systemic and metabolic changes, including acute phase responses to infections [23]. Although many studies focused on tissues directly associated with infection sites [32,38,39,50,53,54,55,56,58,61,62,63], some have explored less invasive alternatives, such as abomasal mucosa biopsies for gene expression profiling [40]. Additionally, a few studies have employed in vitro transcriptomic analyses using blood or peripheral blood mononuclear cells (PBMCs) stimulated with parasite antigens [57,64,65], and ex vivo abomasal ovine models [66] to investigate systemic immune responses.

A condensed overview of RNA-Seq studies in sheep is presented in Table 1, highlighting breed, nematode species, tissue sample type, and infection type. This overview illustrates the limited breed coverage relative to global sheep diversity and emphasizes the need for broader transcriptomic investigations to inform sustainable breeding programs. In contrast, a detailed summary of research studies is provided in Table S1, which includes information on host species (sheep and goat), breed, experimental location, infection type (natural or experimental challenge), tissue sampled, and sampling time point.

Several RNA-Seq studies have examined abomasal transcriptomic responses to *H. contortus* infection in sheep with varying resistance phenotypes. In a comparative transcriptomic analysis, Guo et al. [53] showed that resistant Canaria Hair Sheep Breed (CHB) mounted a stronger response than susceptible Canaria Sheep (CS), with 711 DEGs versus 49 in CS. Upregulated genes in CHB were involved in Th2 cytokine signaling (e.g., *IL10* (interleukin-10), *IL13*), chemokines, complement activation, accelerated cell proliferation, and extracellular matrix remodeling. Selected genes, including *CFI* (complement factor I), *CXCR6* (CXC motif receptor 6), *LGALS15* (lectin, galactoside-binding, soluble, 15), and *MMP1* (matrix metallopeptidase 1), were validated by quantitative reverse transcription PCR (qRT-PCR), confirming the RNA-Seq findings. These results suggest that rapid immune activation, tissue repair, and modulation of parasite fecundity contribute to the resistance in CHB.

Another study by Lins et al. [58] investigated resistant Santa Ines (SI) and susceptible Ile de France (IF) suckling lambs, identifying 292 DEGs in the abomasal mucosa, most of which were upregulated in SI lambs. Integration with single-cell RNA-Seq data suggested that endothelial and tuft cells contributed strongly to resistance, with SI lambs showing higher tuft cell proportions. The study highlighted the interplay between innate and adaptive immunity, particularly via antigen processing and presentation (APP), mainly by T cell APP, macrophage differentiation, and cytokine signaling, providing early-life markers and mechanisms of resistance.

Moreover, El-Ashram et al. [63] analyzed abomasal transcriptomes of sheep at early (7 days post-infection (dpi)) and late (50 dpi) stages of *H. contortus* infection. Early infection stage triggered a pronounced change in gene expression, with 548 genes upregulated and 301 downregulated, whereas late infection stage induced minimal alterations. KEGG pathway analysis identified seven pathways enriched during early infection stage. Upregulated genes in early infection stage were primarily associated with innate immune activation and tissue responses, including *IL-6*, *IL-8*, complement components 1q (*C1q*; initiator of classical complement pathway) and *7* (*C7*; part of the terminal complement pathway involved in membrane attack complex formation), *ACKR3* (atypical chemokine receptor 3), *CCL2* (C-C motif chemokine ligand 2), *MnSOD* (manganese superoxide dismutase), integrin alpha and beta (*ITGA-7/8/9*, *ITGB1/6*), *ICAM-1* (intercellular adhesion molecule-1), and *ACTA-1* (actin, alpha 1, skeletal muscle). In the late infection stage, the transcriptional profile shifted towards inflammation, cytokine signaling, and tissue remodeling. Importantly, *galectin-11* and matricellular protein *osteopontin* were upregulated during chronic infection, whereas *galectin-4* was downregulated during early infection, suggesting these molecules play a role in infection progression and host response dynamics. qRT-PCR validation confirmed the RNA-Seq results, demonstrating high reliability of the results. Overall, the study indicates that early *H. contortus* infection triggers a robust local immune and tissue response, while later stages of infection involve metabolic adaptation and sustained host defense mechanisms.

In Liu et al. [56], RNA-Seq was used to compare abomasal transcriptomic responses 3 days after primary (innate) versus tertiary (acquired) *H. contortus* challenge in resistant and susceptible lines from two Merino flocks (*Haemonchus* selection flock; HSF and *Trichostrongylus* selection flock; TSF) that had been divergently selected for GIN resistance for 40 years. Only 2 and 15 DEGs were detected between primary and tertiary infection in the resistant and susceptible lines of the HSF flock, respectively, whereas 134 and 128 DEGs were identified in the corresponding TSF lines. Across all four resistant and susceptible lines, the *MCP1* gene (mast cell protease 1) was consistently expressed at lower levels in tertiary than in primary infection. In the TSF flock, DEGs in the resistant line were mainly enriched in immune system and B cell receptor signaling pathways, while DEGs in the susceptible line were associated with extracellular matrix and adhesion related terms, suggesting that the development of adaptive immunity to *H. contortus* is highly flock- and line- specific rather than driven by a single conserved DEG signature.

A study by Dixon et al. [23] examined the liver transcriptome of sheep naturally exposed to GINs to identify genes and biological processes regulating the host response. Using RNA-Seq, researchers compared GIN-exposed sheep with either high or low parasite burdens to unexposed control sheep. No significant differential gene expression was detected between high- and low-burden animals, suggesting that liver transcriptional responses were similar regardless of parasite load. In contrast, substantial transcriptomic differences emerged when each exposed group was compared with the control group. Low-burden sheep showed 146 DEGs (64 upregulated, 82 downregulated), while high-burden sheep showed 159 DEGs (57 upregulated, 102 downregulated). Of these, 86 DEGs were shared between both parasite-burden groups relative to controls. Functional enrichment of these shared genes revealed upregulation of immune-related pathways and downregulation of genes associated with lipid metabolism. Several immune-related genes, including *B2M* (beta-2-microglobulin), *OLA-I* (Obg-like ATPase 1), and *CD74* (cluster of differentiation 74), were highly expressed, while acute-phase proteins such as *ORM1* (Orosomucoid 1) and *HP* (haptoglobin) were strongly upregulated, indicating activation of inflammatory pathways. Overall, the study provides insight into hepatic molecular responses during natural GIN exposure and identifies candidate regulatory genes that may contribute to parasite resistance.

In another study, Aboshady et al. [40] used RNA-Seq to investigate time-dependent transcriptomic changes in the abomasal mucosa of goats experimentally infected with *H. contortus*. Biopsies collected at multiple time points revealed dynamic immune responses that differed between resistant and susceptible animals. While both groups exhibited transcriptomic shifts post-infection, resistant goats showed earlier activation of immune-related pathways, including T-cell activation, leukocyte adhesion, and lymphocyte differentiation. Notably, *TGF-β1* (transforming growth factor, beta 1) expression was elevated in resistant goats at 8 dpi but downregulated by 35 dpi, while Th2 and Th17 pathway genes showed sequential activation in resistant animals. This study highlights the importance of early mucosal immune activation in conferring resistance to GIN infection.

Several studies have used RNA-Seq to investigate host transcriptional responses to *T. circumcincta*, one of the most economically important GIN species in temperate regions [38]. These studies typically compare resistant and susceptible sheep to identify DEGs and pathways that regulate protective immunity in the abomasal mucosa and lymph nodes.

In McRae et al. [38], authors examined the transcriptome of the abomasal lymph nodes of Scottish Blackface lambs with divergent resistance phenotypes following an artificial challenge with *T. circumcincta*. Tissues were sampled at 7 and 14 dpi. The study identified 194 DEGs at 7 dpi and 144 DEGs at 14 dpi between resistant and susceptible lambs. Pathway analysis showed that genes involved in the inflammatory response, T lymphocyte recruitment, and leukocyte binding were upregulated in resistant animals at 7 dpi but shifted to higher expression in susceptible animals by 14 dpi. These findings indicate that resistant lambs mount a faster and more effective immune response, whereas susceptible lambs exhibit a delayed activation of key immune pathways. qRT-PCR validation confirmed the RNA-Seq results, demonstrating high reliability of the results.

A study by Chitneedi et al. [32] analyzed abomasal mucosa and lymph node transcriptomes of adult Churra ewes classified as resistant or susceptible based on fecal egg counts. At 7 dpi, RNA-Seq analysis identified 106 GIN-activated DEGs in lymph node tissue. Functional enrichment highlighted cytokine-mediated immune responses, *PPARG* (peroxisome proliferator-activated receptor gamma) signaling, and pathways related to inflammation and gastrointestinal disease. Key resistance-associated genes, including *ITLN2* (intelectin-2), *CLAC1* (laccase domain containing 1), and *galectins*, were confirmed through systematic comparison with previous studies, indicating conserved molecular mechanisms underlying *T. circumcincta* resistance in sheep.

In a study of three-month-old lambs, Canaria Hair sheep (resistant) and Canaria Sheep (susceptible) showed no significant breed-level differences following *T. circumcincta* infection, although substantial individual variability was observed [54]. Analysis of abomasal lymph nodes, mucosal IgA (immunoglobulin A), and local immune cell populations revealed that protection was associated with higher IgA levels, increased numbers of globular leukocytes and major histocompatibility complex class II (MHC-II^+^) cells, and activation of immune pathways including leukocyte adhesion, T cell activation, differentiation, and *IL-4* production. These findings suggest that early-life resistance involves coordinated humoral, cellular, and transcriptional responses [54].

#### 3.2.2. Alternative mRNA Isoform Expression

In mammals, most genes give rise to multiple transcript isoforms or mRNA isoforms through mechanisms such as alternative transcription initiation and termination sites, alternative polyadenylation, and most prominently, alternative splicing [67,68]. More than 90% of human genes have been reported to undergo alternative splicing [69], underscoring their significant contribution to transcriptomic diversity. Importantly, alternative transcript isoforms produced from the same gene can participate in numerous biological processes and metabolic pathways [70,71], and disruptions in their expression have been linked to various diseases, including cancer [68].

RNA-Seq has become central to the identification and quantification of mRNA isoforms at high-throughput level. It also enables the detection of known and novel splice junctions, characterization of isoform structure, and accurate estimation of isoform abundance [72,73]. Consequently, mRNA isoform-level RNA-Seq analyses can offer a powerful approach to uncover alternative splicing events that may contribute to differential immune responses in GIN infected sheep. Nevertheless, no studies to date have investigated mRNA isoform-level expression to understand the molecular mechanisms underlying genetic resistance to GIN infections in sheep.

#### 3.2.3. Differential Long Non-Coding RNAs Expression

LncRNAs are non-coding RNA molecules longer than 200 nucleotides. Despite their structural similarity to mRNAs, they typically do not encode proteins [74,75]. They represent a diverse class of transcripts with many functions still being uncovered. Based on the current understanding, functions of lncRNAs can be classified into three groups: (1) non-functional transcripts, (2) regulators of transcription that modulate the expression of protein-coding genes through cis- or trans-acting mechanisms [75], and (3) post-transcriptional regulators involved in processes such as alternative splicing, translational control, and competition with endogenous regulatory RNAs [51,76].

Studies have shown that lncRNAs exhibit features commonly seen in protein-coding genes, including cell- and tissue-specific expression patterns [51,77] as well as the presence of splice variants and introns [76]. Owing to their regulatory roles and highly specific expression profiles, lncRNAs have gained attention as potential epigenetic biomarkers, particularly in disease diagnostics and prognostics [77]. Recent studies in mice [78] and humans [79] have shown that lncRNAs play a role in modulating the immune response to parasitic infections. Nonetheless, there is limited evidence regarding the role of lncRNAs in regulating immune responses in sheep during GIN infections.

Evidence of lncRNA involvement in the host immune response to GIN infection in adult Churra sheep has been reported in a recent RNA-Seq study by Chitneedi et al. [51]. Using abomasal lymph node tissue from sheep differing in resistance to *T. circumcincta*, the authors identified 9105 putative lncRNA transcripts, among which 457 loci encompassing 683 lncRNAs were differentially expressed between resistant and susceptible animals. Co-expression network analyses revealed that several of these DE lncRNAs were strongly associated with gene network modules enriched for key immune-related pathways, including *ERK5* (extracellular signal-related kinase) signaling, *SAPK/JNK* (stress-activated protein kinases) signaling, oxidative phosphorylation, and translation-related pathways such as *EIF4* (eukaryotic translation initiation factors 4) and *p70S6K* (70 kDa ribosomal protein S6 kinase) signaling. The *EIF4* and *p70S6K* signaling pathways regulate cell growth and stress responses by promoting protein biosynthesis through control of the translational machinery. Collectively, these findings suggest that DE lncRNAs may act as potential regulatory elements influencing stress responses, immune signaling, and metabolic activity during GIN infection.

#### 3.2.4. Functional Genetic Variants

The discovery of functional genetic variants underlying traits of interest is a cornerstone of livestock genetics studies. SNPs, which are single-base variations found in coding, non-coding, and regulatory regions, can significantly impact protein function and gene expression [80]. High-throughput RNA-Seq enables the detection of SNPs across expressed regions of the genome in various tissues and species [45,46,48,49]. RNA-Seq-based variant detection has been shown to be a reliable and cost-effective alternative to whole-genome or whole-exome sequencing, with over 98% of SNPs identified by RNA-Seq also detected by these approaches [47].

To date, no studies have reported the discovery of SNPs specifically using RNA-Seq data derived from GIN-infected tissues. Although Aboshady et al. [50] and Chitneedi et al. [25] investigated functional genetic variants in ovine abomasal lymph nodes, including SNPs and INDELs, collectively as a single set of variants and performed functional annotation for all variants together, as discussed in the functional INDELs section of this review.

INDELs are an important yet underexplored class of genetic variations that can be detected from RNA-Seq data, with potential effects on resistance or susceptibility to GIN infections in sheep. The RNA splicing process is a critical regulator of gene expression, and variants that disrupt normal splicing are a well-established cause of diseases [81]. While the search for such variants has historically focused on single nucleotide variants (SNVs), there is growing recognition that insertions and deletions (INDELs) are a significant source of splicing abnormalities or generating novel splice motifs, leading to exon extension or shrinkage events [81]. Advances in high-throughput RNA-Seq allow the identification of expressed functional and structural variants, including INDELs, in addition to quantifying gene expression [82]. In Yang et al. [83], authors demonstrated the clinical relevance of INDELs by linking them directly to human diseases using the transIndel tool. Ultimately, the identification of such variants can map causal mutations for complex traits and serve as molecular markers in genomic evaluations, enhancing the accuracy of selection in genetic improvement programs [3,48].

Notably, Cunha et al. [3] identified functional INDELs affecting splice sites in Rideau x Dorset crossbred sheep naturally exposed to GIN infections, using RNA-seq of liver tissue from animals exhibiting distinct innate immune profiles. Lambs with high stress responder (HSR) and medium stress responder (MSR) profiles (GIN-exposed), along with unexposed controls, were analyzed. The study identified 34,168 and 39,380 INDELs in HSR and MSR animals, respectively, of which 1614 (HSR) and 2980 (MSR) were predicted to impact splice sites. These splice-site-associated INDELs mapped to 514 genes in HSR and 855 genes in MSR, including key immune-related genes such as *STAT6* (signal transducer and activator of transcription 6) and *CFI* (complement factor I). These findings highlight that INDELs can modulate splice-sites and influence transcript structure, contributing to phenotypic variation in host resistance and identifying potential targets for genomic selection.

Moreover, a complementary study by Aboshady et al. [50] investigated genomic variants in the abomasal mucosa of Creole goats experimentally infected with *H. contortus* GIN, comparing resistant and susceptible phenotypes using RNA-Seq. Resistant animals exhibited a substantially higher number of unique genetic variants than susceptible counterparts, including SNPs (154,545 vs. 53,165), insertions (11,708 vs. 6890) and deletions (10,302 vs. 4746). Functional analysis of combined variants (SNPs and INDELs) showed significant enrichment in immune-related pathways in resistant animals, notably the *MAPK* (mitogen-activated protein kinase) signaling pathway, T cell receptor (TCR) signaling pathway, hepatitis B, and longevity-regulating pathways. These pathways were not enriched in susceptible animals, highlighting their potential role in modulating host immune responses to GIN infections.

To further characterize the genetic basis of resistance to *T. circumcincta*, Chitneedi et al. [25] performed RNA-Seq based variant discovery on lymph node tissue from six resistant and six susceptible adult Churra dairy ewes following controlled experimental infection. Using a stringent variant-calling pipeline that retained only high-confidence variants identified by both Genome Analysis Toolkit (GATK) and Sequence Alignment/Map Tools (SAMTOOLS), the authors examined two biologically relevant target sets: previously reported GIN-resistance QTL and functional candidate genes identified through gene expression studies. They identified 930 genes within QTL regions and 553 candidate genes harboring putative functional variants, including 111 and 132 immune-related genes, respectively. Enrichment analyses of QTL-related variants were associated with biological processes such as apoptosis, cell adhesion, and inflammatory response. In contrast, variants in candidate GIN-activated genes were linked to disease-related processes including inflammation, adhesion, and necrosis. Hence, this study provided a targeted catalogue of potential causal variants that may underlie genetic resistance to GIN infections, highlighting promising candidate variants for integration into breeding programs aimed at improving parasite resistance.

Across multiple studies, transcriptomic studies demonstrated that resistance to *H. contortus* and *T. circumcincta* in sheep is associated with a rapid and coordinated immune response, particularly at early stages of infection. The most consistently observed mechanisms include activation of Th2 cytokine signaling (*IL4*, *IL10*, *IL13*), chemokines, complement components (*C1q*, *C7*, *CFI*), antigen processing and presentation, and tissue repair/remodeling. Resistant animals also showed early recruitment and activation of T cells, macrophages, and tuft cells, along with higher local IgA levels, indicating an interplay of innate and adaptive immunity. Emerging evidence further suggests that lncRNAs act as regulatory modulators of immune and stress-response pathways, while functional genetic variants, including SNPs and INDELs affecting splice sites, influence gene expression and transcript structure in key immune genes (*STAT6*, *CFI*, *MAPK*, and *TCR* signaling components). Across breeds, these patterns highlight that early and effective local immune activation, complement-mediated defense, antigen presentation, and coordinated tissue responses are the most robust transcriptomic markers of GIN resistance.

## 4. Genomic Landscape in Host–Parasite Interactions

Understanding the genetic basis of host resistance to GIN has become increasingly important as conventional control strategies have become less effective and natural infections remain highly complex in field settings. Although phenotypic indicators such as FEC, antibody levels, Faffa Malan Chart (FAMACHA) score (a conjunctival color-based anemia scoring system used to assess parasite burden in small ruminants), and packed cell volume (PCV) have long been used to identify resistant animals [2,84,85], these measures capture only a fraction of the underlying phenotypic variation, and the mechanisms driving genetic variation in resistance remain poorly defined [86]. Numerous studies have reported both between- and within-breed variation in GIN resistance in small ruminants [87,88] and observed differences in immune responses among GIN-infected individuals further support a genetic basis of resistance [23,38]. Recent advances in genomic technologies, particularly GWAS [89], provide powerful tools to dissect the genetic variation underlying complex and polygenic traits [86]. Identifying candidate genes, regions, and regulatory genetic variants associated with GIN resistance can enhance our understanding of the molecular mechanisms involved and facilitate accelerated genetic improvement in breeding programs. Several studies have reported that SNP based GWAS have revealed multiple candidate genes, regions, and QTL linked to GIN resistance across nearly all ovine chromosomes [86].

In Ahbara et al. [86], genome-wide selection signatures analyses were performed in Tunisian sheep genotyped with a 600 K SNP array to identify regions associated with GIN resistance. By integrating multiple genomic analyses, runs of homozygosity, LR-GWAS (logistic regression-GWAS), FST (fixation index), and XP-EHH (cross-population extended haplotype homozygosity), the author identified 35 candidate regions across 19 autosomes, spanning 121 genes. Nineteen of these regions overlapped with QTL for immune function, parasite resistance, and disease susceptibility traits. Candidate genes within these regions included those involved in innate immune defense (solute carrier family 22 member 4 and 5 (*SLC22A4* and *SLC22A5*), *IL4*, *IL13*), intestinal wound healing (*VIL1*; villin 1, *CXCR1*, *CXCR2*), and GIN expulsion (*IL4*, *IL13*). Functional enrichment analyses of candidate genes highlighted pathways related to immune system processes, cytokine receptor interactions, intestinal innate immunity, and integrin signaling, supporting their role in host defense against GIN infection.

Similarly, Thorne et al. [90] conducted GWAS in Rambouillet and Dorper × White Dorper lambs to identify SNPs associated with GIN resistance, using FEC and PCV as phenotype indicators during *H. contortus* infection. The analysis identified 26 significant SNPs, 21 of which were mapped to genes including *SCUBE1* (signal peptide, CUB domain and EGF like domain containing 1), *GALNT6* (polypeptide N-acetylgalactosaminyltransferase 6), *IGF1R* (insulin like growth factor 1 receptor), *CAPZB* (capping actin protein muscle Z-line beta subunit), and *PTK2B* (protein tyrosine kinase 2 beta), which are involved in immune cell development, mucin production, and epithelial wound healing, highlighting key pathways in the host response to *H. contortus* infection. These findings collectively demonstrate the potential of genomic approaches to improve sheep resistance to GIN.

A large-scale single-step GWAS (ssGWAS) conducted in Corriedale sheep by Carracelas et al. [91] further demonstrated the polygenic nature of GIN resistance and highlighted several genomic regions contributing to variation in FEC. The study used an extensive dataset of 19,547 lambs with FEC phenotypes, a pedigree of 40,056 animals, and genotypes obtained from three SNP panels (170 SNPs, 507 SNP chip, and 50 K SNP chip). Using chromosome-wise false discovery rate thresholds, the authors identified multiple significant regions across the ovine genome, although the detected loci varied by SNP density. The 170-SNP panel revealed associations on chromosomes 1, 3, 12, and 19, pointing to candidate genes such as *TIMP3* (TIMP metallopeptidase inhibitor 3), toll-like receptor 5 and 9 (*TLR5*, *TLR9*) and *LEPR* (leptin receptor), all of which have roles in innate immunity, inflammation, or tissue remodeling. The 507-SNP chip identified regions on chromosomes 7, 12, and 24, including *SYNDIG1L* (synapse differentiation inducing 1 like) and *MGRN1* (mahogunin ring finger 1), while the 50 K SNP chip detected significant signals only on chromosome 7, highlighting genes such as *INO80* (INO80 complex ATPase subunit), *TLN2* (talin 2), *TSHR* (thyroid stimulating hormone receptor), and *EIF2AK4* (eukaryotic translation initiation factor 2 alpha kinase 4). Many of these loci overlap previously reported QTL for FEC in Corriedale and other breeds, reinforcing their relevance to host resistance. Importantly, the discovery of novel candidate genes, particularly those involved in immune signaling, receptor activity, and cellular stress response, expands the genomic landscape influencing parasite resistance.

In addition to these genome-wide studies, targeted sequencing approaches have provided complementary insights into the genetic basis of resistance. In Estrada-Reyes et al. [92], researchers evaluated 153 Florida Naïve lambs using a custom panel of 100 immune-related genes to identify SNPs associated with natural *H. contortus* exposure. Eighteen significant SNPs were detected across 12 chromosomes, mapping to genes involved in Th17, Treg, and Th2 immune pathways, including *STAT3*, *STAT6*, *IL2RB* (interleukin 2 receptor subunit beta), *CXCL10*, *TNF* (tumor necrosis factor), *TLR3*, *ITGA4* (integrin subunit alpha 4), and *MUC15* (mucin 15, cell surface associated) genes. These genes play essential roles in cytokine signaling, immune regulation, mucosal defense, and inflammatory responses. These findings further demonstrate that genetic variation in key immune pathways contributes significantly to the expression of resistant phenotypes in sheep.

Overall, genomic studies highlight that resistance to GIN infections in sheep is a highly polygenic trait, with multiple loci and genes consistently associated with immune regulation, mucosal defense, and tissue repair. Across breeds and studies, the most robustly supported mechanisms include: cytokine signaling (Th2 and Th17 pathways, e.g., *IL4*, *IL13*, *STAT3*, *STAT6*), innate immune recognition (*TLRs*, *CXCR1/2*), epithelial and mucosal integrity (*VIL1*, *MUC15*, *TIMP3*), and cellular stress or signaling pathways (*LEPR*, *EIF2AK4*, *SYNDIG1L*). Both genome-wide and targeted approaches converge on these pathways, emphasizing that genetic variation in immune activation, pathogen recognition, and tissue remodeling underlies phenotypic resistance.

## 5. Metabolome and Proteome Landscape in Host–Parasite Interactions

In relation to the metabolomic alterations observed in the host’s response to GIN infection, Hempstead et al. [93] conducted an investigation into systemic metabolic changes in lambs utilizing a trickle infection model. Their lipidomic analysis revealed elevated plasma concentrations of specific triglycerides, phosphatidylcholines, and sphingomyelins in challenged lambs by the third week of infection, prior to peak FECs or observable reductions in liveweight gain. Notably, the triglycerides that differed all contained at least one highly unsaturated fatty acid, typically corresponding to an n-3 fatty acid, with the exception of one ether-triglyceride. All of the differing fatty acids were elevated in the challenged lambs.

A further investigation conducted by Xiang et al. [94] examined region-specific amino acid responses to *H. contortus* infection in sheep in the abomasum, duodenum, and skeletal muscle. The analysis indicated significant changes in 36 and 19 metabolites found in the abomasal and duodenal chyme, respectively. The infection was found to enhance the metabolism of arginine and sulfur-containing amino acids (such as cysteine and methionine) in the abomasum, while simultaneously decreasing the metabolism of pyruvate-related amino acids (including alanine and serine) in the duodenum. In skeletal muscle, there was a noted reduction in the concentrations of arginine, histidine, and cysteine, whereas levels of glycine and alanine were found to be elevated. Those results underscore the influence of GIN infection on the host’s amino acid and lipid metabolism, indicating a metabolic shift that favors localized defense mechanisms and tissue maintenance, which may serve as potential biomarkers of GIN parasitism.

In addition to metabolomic insights, proteomic approaches have further expanded our understanding of host responses to GIN infection. The study by Chagas et al. [33] utilized liquid chromatography-tandem mass spectrometry (LC-MS/MS) based serum proteomics to examine the differences in protein abundance between resistant (Santa Inês) and susceptible (Dorper) sheep breeds during the course of infection. Among the 754 proteins identified, 68 exhibited differential abundance, which included acute phase proteins and immune-related factors. Notably, phosphopyruvate hydratase (ENO3) showed consistent differences across all comparisons, indicating its significant role in breed-specific immune or metabolic responses. The proteins that were upregulated in infected animals were mainly linked to immune activation and inflammation, whereas the downregulated proteins suggested a reduction in tissue anabolism and possible disruptions in muscle and fat metabolism. The proteome of gastric lymph was also studied in sheep to evaluate localized immune responses to *T. circumcincta* infection [95]. Proteomic analysis of this lymph revealed significant infection-induced changes in proteins such as gelsolin, α-1B glycoprotein, and haemopexin. Other tissues have also been investigated using proteomics, such as abomasal mucosa [96] and intestinal lymph [97], revealing tissue-specific responses.

In contrast to research examining the metabolic responses of hosts to GIN infection, Godoy et al. [98] analyzed the exo-metabolome of adult *H. contortus* worms recovered from infected lambs. Utilizing untargeted lipidomics of the culture media, they discovered a wide variety of secreted lipid species, such as phosphatidylcholines, sphingomyelins, and ether lipids. Similar in vitro study [99] detected lysophosphatidylglycerols, diglycerides, fatty acyls, glycerophospholipids, and a triglyceride in the secretome of *H. contortus*. Those studies underscore the intricate metabolome profile of the parasite itself, which may be involved in immune modulation, nutrient acquisition, or signaling between the host and the parasite. In conjunction with metabolomic studies centered on the host, these results further our comprehensive understanding of the biochemical interactions that are fundamental to GIN infections. By analyzing the lipidome that is excreted and secreted by parasitic nematodes, this research also improves the feasibility of studying these organisms in relation to small-molecule interactions pertinent to pathogenesis, vaccine and drug development, as well as the identification of metabolic biomarkers derived from parasites.

Taken together, metabolomic and proteomic studies indicate that *H. contortus* and *T. circumcincta* infections trigger consistent shifts in host lipid and amino acid metabolism, as well as in immune-related protein abundance. Key host responses include elevated unsaturated triglycerides and phospholipids, enhanced metabolism of arginine and sulfur-containing amino acids, and reduced pyruvate-related amino acids, reflecting a metabolic reprogramming that supports tissue maintenance and localized defense. Proteomic analyses highlight upregulation of acute-phase and immune proteins (ENO3, gelsolin, α-1B glycoprotein) and downregulation of anabolic pathways. These patterns suggest that coordinated metabolic and proteomic responses, particularly involving energy allocation, amino acid utilization, and immune protein induction, are robust markers of host resistance and potential biomarkers for monitoring or enhancing parasite resilience.

## 6. Microbiome Landscape in Host–Parasite Interactions

The microbiome represents an additional omics technology that has been extensively researched in the context of GIN infections in sheep. In Corrêa et al. [100], authors investigated lambs mixed-infected with *H. contortus* and *Trichostrongylus colubriformis*, which exhibited reduced abundance of butyrate-producing bacteria and shifts in microbial metabolic pathways in the rumen, including those related to nitrogen and amino acid metabolism. While tannin supplementation (*Acacia mearnsii*) influenced microbial diversity, the infection itself was a major driver of microbiome structural changes. In another study, Cortés et al. [35] assessed the effects of *T. circumcincta* infection on the gut microbiome and immune environment in sheep. According to the results found, alterations in microbial composition were mainly influenced by parasite infections instead of vaccinations, highlighting a significant increase in bacterial groups linked to pro-inflammatory responses. In particular, certain taxa, including *Prevotella*, *Porphyromonas*, and *Sutterella*, were identified as contributors to abomasal inflammation, indicating their involvement in the development of parasitic gastroenteritis. Rooney et al. [36] corroborate these results, showing that vaccination against *T. circumcincta* also impacted the gut microbiome of lambs, highlighting that *Prevotella* spp. was significantly linked to helminth infection.

In an effort to further elucidate the dynamics of host–microbiota–helminth interactions, Paz et al. [34] conducted a comparative analysis of the gastrointestinal microbiota in both helminth-resistant and -susceptible sheep, utilizing 16S rRNA gene sequencing across various regions of the gut. Notable differences were identified between the two groups, especially in the duodenum, where the resistant sheep demonstrated enhanced microbial diversity and a higher abundance of carbohydrate-fermenting genera, including *Aminipila*, *Lachnoclostridium*, and *Mogibacterium*. These findings indicate that resistant sheep possess unique microbial profiles throughout the gastrointestinal tract, implying that alterations in the duodenal microbiota may contribute to the inhibition of helminth development.

Other studies have investigated the impact of GIN infection on the microbiome in sheep [101,102,103], demonstrating GIN- and tissue-specific modulation of metacommunities. Such evidence highlights the potential role of microbiota to the immunopathology of GIN infections.

Collectively, microbiome studies indicate that GIN infections consistently alter gut microbial composition and metabolic activity, with resistant sheep exhibiting higher microbial diversity and increased abundance of carbohydrate-fermenting taxa in the duodenum. In contrast, susceptible or infected animals show proliferation of pro-inflammatory-associated taxa such as *Prevotella*, *Porphyromonas*, and *Sutterella*. These findings suggest that a diverse, functionally supportive microbiome may contribute to helminth resistance by promoting gut homeostasis, modulating inflammation, and potentially limiting parasite development, highlighting specific microbial signatures as robust markers of host resistance.

## 7. Integrative Multi-OMICs Approaches in Host–Parasite Interactions

Advances in high-throughput technologies and declining sequencing costs now enable the generation of large-scale datasets across multiple omics layers, including transcriptomics, genomics, metabolomics, proteomics and microbiomics [104]. While each omics technology individually contributes valuable insights, their integration provides a far more comprehensive understanding of the molecular mechanisms underlying complex traits and diseases [105]. Multi-omics integration allows researchers to uncover novel associations between biomolecules and phenotypes, identify key regulatory signaling pathways, and establish biomarkers that influence a particular trait [106]. Applied to parasitic infections in sheep, the integration of multi-omics data is a powerful strategy for elucidating the molecular mechanisms underlying immune responses and host–parasite interactions [29] that drive variation in resistance and susceptibility within and between breeds [27].

**Figure 1:**
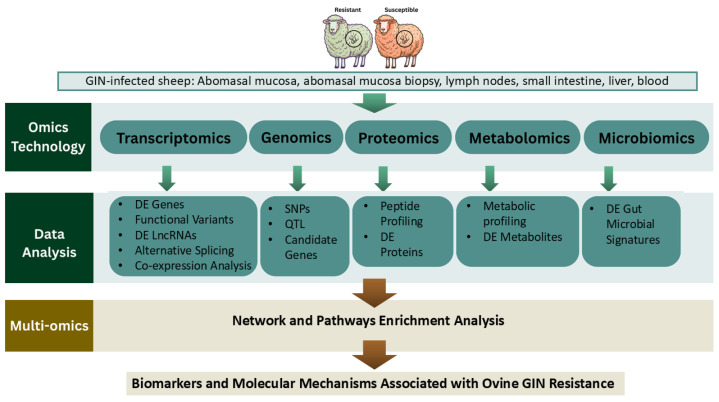
Omics strategies for studying genetic resistance to GIN infections in sheep.

The workflow of multi-OMICs analysis for genetic resistance to GIN infections in sheep is outlined in Figure 1. Multi-omics approaches also provide a systems-level framework for dissecting the complex biological processes underlying GIN resistance. Transcriptomics, genomics, proteomics, metabolomics, and microbiomics capture distinct layers of host biology, and their integration enables the identification of regulatory networks and biomarkers that cannot be detected using single omics approaches alone. In GIN-infected sheep, transcriptomic changes reflect immune activation, genomic variants help pinpoint causal loci, while proteomic and metabolomic shifts reveal downstream functional and physiological responses. Linking these datasets through correlation analyses, pathway mapping, and network construction allows researchers to connect molecular mechanisms across omics layers to resistance phenotypes and identify robust regulatory pathways that underpin variation in host resistance.

A recent integrative multi-OMICs study in Morada Nova sheep infected with *H. contortus* combined differential gene expression analysis, RNA-seq variant calling, GWAS, and gene–microbiome co-expression network analysis to identify molecular mechanisms underlying parasite resistance [27]. In the abomasal transcriptome, 11 genes were differentially expressed between resistant and susceptible animals, including *GAST* (gastrin), *IL13*, and *GNLY* (granulysin), implicating immune modulation, gastric function, and host adaptation. Variant calling of RNA-seq data revealed SNPs in *MGRN1*, *TRAPPC6B* (trafficking protein particle complex subunit 6B), and *SPCS3* (signal peptidase complex subunit 3), with *MGRN1* gene previously associated with nematode resistance. GWAS identified significant SNPs on chromosomes 2 and 11, linked to candidate genes involved in immune responses, adaptation, and microbiota regulation, such as *FGF14* (fibroblast growth factor 14) and *RORC* (RAR related orphan receptor C). Co-expression network analysis further highlighted hub genes (*CD8A*, *CD8B*, *TRGC1*; T cell receptor gamma constant 1, *CALHM6*; calcium homeostasis modulator family member 6) and microbial taxa (*Christensenellaceae*, *Bacteroides*, *Prevotella*, *Methanobrevibacter*) associated with resistance, demonstrating the interplay between host immune genes and gut microbiota.

Multi-omics studies show that resistance to GIN infections in sheep is underpinned by coordinated immune and physiological mechanisms detectable across omics layers. Transcriptomic and proteomic data highlight early immune activation, including Th2 cytokine signaling (*IL13*), T cell receptor genes (*CD8A/B*, *TRGC1*), and antimicrobial effectors (*GNLY*). Genomic analyses identify SNPs in immune and adaptation related genes (*MGRN1*, *FGF14*, *RORC*), while metabolomic and microbiome profiling reveal complementary shifts in host metabolism and gut microbial composition (*Christensenellaceae*, *Bacteroides*, *Prevotella*) that correlate with resistance.

## 8. Conclusions

Conventional GIN control strategies are increasingly challenged by widespread anthelmintic resistance, underscoring the need for sustainable alternatives. Genetic resistance offers a promising strategy to improve host resistance while reducing parasite burden. This review synthesizes current evidence from transcriptomics and complementary omics technologies to elucidate the molecular and genetic mechanisms underlying host resistance to *H. contortus* and *T. circumcincta* infections in sheep.

Transcriptomic studies across different post-infection time points and key tissues such as the abomasum, lymph nodes, and liver have revealed time-dependent and tissue-specific immune signatures. Early responses are dominated by innate and Th2-associated pathways, including *IL4/IL13* signaling, *STAT6* activation, chemokines, complement components, and *galectins*, whereas later stages involve tissue repair, extracellular matrix remodeling, metabolic adaptations, and mechanisms of parasite expulsion or persistence.

Substantial breed variation further shapes resistance phenotypes. While conserved immune pathways are shared across breeds, distinct transcriptional, genomic, proteomic, and metabolomic profiles reflect differences in genetic background, selection history, and production environments. Transcriptomic analyses have also revealed lncRNAs and functional genetic variants, including SNPs and INDELs in expressed regions and splice sites, as important regulatory contributors to resistance. Genomic studies corroborate the polygenic nature of GIN resistance, identifying multiple SNPs, QTLs, and candidate genes linked to immune regulation, intestinal integrity, and parasite control. Proteomic and metabolomic studies reveal downstream functional consequences of infection and resistance, while microbiome analyses indicate that resistant sheep exhibit distinct gastrointestinal microbial communities that may enhance gut homeostasis and limit parasite establishment. Integrative multi-omics approaches further illuminate the interactions between host genetics, tissue-specific regulation, metabolic pathways, and the gastrointestinal microbiome.

Notably, the lack of mRNA isoform-level expression analyses remains a key knowledge gap. Future research combining multi-omics, functional annotation, and host–microbiome interactions across breeds will refine candidate biomarkers and guide targeted breeding strategies, supporting the development of sustainable, parasite-resistant sheep flocks and reducing reliance on chemical treatments across global sheep production systems.

## Figures and Tables

**Table 1:** Summary of RNA-Seq studies on gastrointestinal nematode resistance in sheep.

Breed	Nematode Species		Tissue Sample Type				Infection Type		Reference
	*HC*	*TC*	AM	ALN	Blood/PBMC	Liver	EI	N	
Canaria Hair and Canaria Sheep	✓		✓				✓		[53]
Scottish Blackface		✓		✓			✓		[38]
Churra		✓	✓	✓			✓		[32]
Merino (HSF and TSF lines)	✓		✓				✓		[39,55,56]
St. Croix and Suffolk	✓				✓				[57]
Canaria Hair and Canaria Blanca		✓		✓			✓		[54]
Santa Ines and Ile de France	✓		✓				✓		[58]
Rideau × Dorset	✓					✓		✓	[4,23]

Note: *HC* = *Haemonchus contortus*; *TC* = *Teladorsagia circumcincta*; AM = Abomasal mucosa; ALN = Abomasal lymph nodes; EI = Experimental infection; N = Natural; HSF = *Haemonchus* selection flock; TSF = *Trichostrongylus* selection flock. Additional breed-specific phenotypic information is in the Supplementary Table S1.

## Data Availability

No new data were created or analyzed in this study.

## Supplementary Materials

The following supporting information can be downloaded at: https://www.mdpi.com/article/10.3390/pathogens15010106/s1, Table S1: Summary of RNA-seq studies on GIN resistance in sheep and goats (from 2015–2025).

## Abbreviations

The following abbreviations are used in this manuscript:

RNA-seq	RNA Sequencing
mRNA	Messenger RNA
GWAS	Genome-wide Association Study
SNPs	Single Nucleotide Polymorphisms
INDELs	Insertions and Deletions
ncRNA	Non-coding RNA
lncRNA	Long non-coding RNA
qRT-PCR	Quantitative Reverse Transcription Polymerase Chain Reaction
LC-MS/MS	Liquid Chromatography-tandem Mass Spectrometry
